# Long-term survival after female pelvic organ-sparing radical cystectomy versus standard radical cystectomy: a multi-institutional propensity score-matched analysis

**DOI:** 10.1097/JS9.0000000000000516

**Published:** 2023-06-16

**Authors:** Wenlong Zhong, Kun Xia, Libo Liu, Sida Cheng, Peng Hong, Wang He, Wen Dong, Hao Liu, Yiming Lai, Han Hao, Cheng Liu, Hongxian Zhang, Xinfei Li, Guangpu Ding, Xuesong Li, Lulin Ma, Liqun Zhou, Tianxin Lin, Jian Huang

**Affiliations:** a Department of Urology, Sun Yat-sen Memorial Hospital; Guangdong Provincial Key Laboratory of Malignant Tumor Epigenetics and Gene Regulation; Guangdong Provincial Clinical Research Center for Urological Diseases, Sun Yat-sen (Zhongshan) University, Guangzhou, PR China; b Department of Urology, Peking University First Hospital, Beijing, PR China; c Department of Urology, Peking University Third Hospital, Beijing, PR China; d Department of Urology, Jiangxi provincial People’s Hospital, The First Affiliated Hospital of Nanchang Medical College, Nanchang, PR China

**Keywords:** bladder cancer, female, pelvic organ preserving-radical cystectomy, radical cystectomy, survival

## Abstract

**Background:**

The application of pelvic organ preserving-radical cystectomy (POPRC) in female patients with bladder cancer has attracted more and more attention in recent years. In the current study, the authors aim to compare the long-term oncological outcomes of POPRC versus standard radical cystectomy (SRC) in a large multicenter retrospective cohort.

**Patients and methods:**

Data on female patients with bladder cancer who underwent POPRC or SRC in January 2006 and April 2018 were included from three Chinese urological centers. The primary outcome was overall survival (OS). Secondary outcomes were cancer-specific survival and recurrence-free survival. To decrease the effect of unmeasured confounders associated with treatment selection, 1:1 propensity score matching was performed.

**Results:**

Among the 273 enrolled patients, 158 underwent POPRC (57.9%), and 115 underwent SRC (42.1%). The median follow-up time was 38.6 (15.9–62.5) months. After propensity score matching, each cohort included 99 matched patients. The OS (*P*=0.940), cancer-specific survival (*P*=0.957), and recurrence-free survival (*P*=0.476) did not differ significantly from the two matched cohorts. Subgroup analysis confirmed that the OS was similar between the patients treated with POPRC and SRC across all subgroups examined (all *P* > 0.05). In multivariable analysis, the surgical method (SRC vs. POPRC) was not an independent risk factor for OS (Hazard ratio 0.874, 95% CI 0.592–1.290; *P*=0.498).

**Conclusions:**

The results showed that no significant difference in long-term survival was determined between female patients undergoing SRC and those undergoing POPRC.

## Introduction

Highlights

- Pelvic organ preserving-radical cystectomy (RC) is oncologically safe in selected female patients.
- Pelvic organ preserving-RC is feasible for elderly patients.
- The concomitant primary genital cancers in women with bladder cancer is rare.
- Genital recurrence is rare in patients receiving pelvic organ preserving-RC.

Bladder cancer is one of the most common malignancies worldwide; however, the morbidity varies between sex with the incidence of 2.2/100 000 per year in female patients^1,2^. Muscle-invasive bladder cancer (MIBC) comprises about 25% of bladder cancer, and radical cystectomy (RC) is the gold-standard treatment for patients with MIBC and high-risk nonmuscle invasive bladder cancer. Compared with male patients, female patients have more muscle-invasive tumors at the time of diagnosis and thus have a worse prognosis^3,4^. For standard radical cystectomy (SRC) in females, the uterus, fallopian tubes, ovaries, anterior vaginal wall, and regional lymph nodes undergo en bloc resection with the bladder and urethra^5^. With regard to the wide range of resection, sexual dysfunction, and continence disorder are very common after surgery. It was reported that 20–89%, 15–41%, and 11–55% of patients suffered from decreased desire, poor vaginal lubrication, and dyspareunia in sexually active patients receiving SRC, respectively^6,7^. The incontinence rate following SRC ranged from 34 to 69% in women receiving orthotopic neobladder reconstruction^8–11^. Thus, the quality of life is severely reduced after SRC, particularly for young patients^12^.

These problems can be addressed by pelvic organ-sparing radical cystectomy (POPRC) for females, which emphasizes the potential benefits of preserving reproductive organs and their neural structure incontinence and urinary retention^13^. POPRC involves the preservation of the female vagina, uterus, neurovascular bundles, and any enhancement of the aforementioned techniques^14^. Previous studies demonstrated that POPRC might not affect long-term survival and instead improve sexual function and continence outcomes^12^. However, most of the available studies are retrospective with limited cases in a single-center. Data regarding long-term oncologic outcomes are sparse.

The current study primarily aims to compare the long-term survival of POPRC versus SRC in a large, multicenter retrospective cohort. This study also intends to investigate the potential indications of POPRC for female patients.

## Patients and methods

### Study population

We retrospectively reviewed charts from 2287 patients who underwent RC in three participating centers from January 2006 to April 2018. The data were obtained from the network database of the Chinese Bladder Cancer Consortium. All female patients with bladder cancer who underwent RC for curative treatment (including high-risk nonmuscle invasive bladder cancer and MIBC) were included. This study was approved by the institutional review board of each participating center. Written informed consent was obtained from each enrolled patient. The work has been reported in line with the strengthening the reporting of cohort, cross-sectional and case-control studies in surgery (STROCSS) criteria^15^, Supplemental Digital Content 1, http://links.lww.com/JS9/A711.

### Patient selection

**Figure 1:**
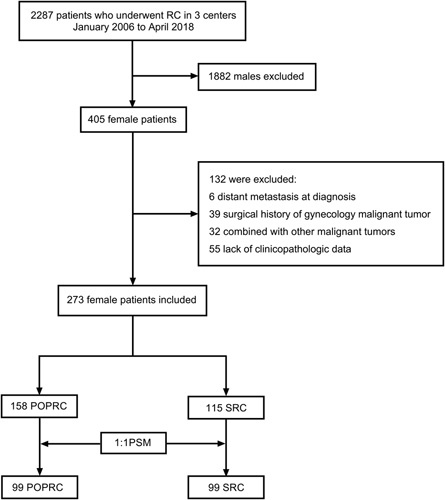
The flowchart of patient selection. RC radical cystectomy, POPRC pelvic organ preserving-radical cystectomy, SRC standard radical cystectomy, PSM propensity score matching.

A flowchart of patient selection is presented in Figure 1. All patients were evaluated for gynecological tumors preoperatively through pelvic MRI or CT. Consensus was reached that the indications of POPRC were as follows: no suspicion of reproductive organs invasion at diagnosis or intraoperatively; without primary genital malignant tumors; without tumors at the urethra bladder neck and trigone; with a strong desire to preserve reproductive organs. The exclusion criteria were as follows: distant metastasis at diagnosis; surgical history of gynecology malignant tumors; concomitant with other malignant tumors; a lack of clinicopathologic data. Surgical methods were classified into either SRC or POPRC, which was agreed upon by the patient and the surgeon.

### Surgical technique for SRC

SRC was performed with the bladder, uterus, fallopian tubes, ovaries, and anterior vaginal wall removed en bloc after bilateral pelvic lymphadenectomy, which involved the resection of lymph nodes around the common, external, and internal iliac vessels, and the obturator vessels. The peritoneum was incised in the Douglas cavity below the vaginal fundus. After approaching the bladder neck, the vaginal wall was opened circumferentially. The vaginal wall was closed transversely by folding the pouch of Douglas down to the caudal resection line along the anterior vaginal wall. As for urinary diversion, ureterocutaneostomy, ileal conduit, and orthotopic ileal neobladder were performed in an extraperitoneal manner.

### Surgical technique for POPRC

The techniques of POPRC were detailed in a previous study by Bhatta Dhar N *et al.* in 2007^13^. Bilateral pelvic lymphadenectomy was performed first consistent with that in SRC. The superior and inferior vessels of the bladder were ligated and transected at the beginning from the internal iliac vessels. The peritoneum was incised at the level of the utero-vesicle junction to develop a vesicle-vaginal plane for the purpose of uterine preservation. The vaginal wall at the cervical level was dissected in the anterior vagina plane of the vagina at the 2 and 10 o’clock position for the purpose of preserving the utero-vaginal and pararectal components of the pelvic plexus. The urethra was removed ~5 mm distal to the vesicourethral junction combined with the bladder. The urinary diversion was consistent with that in SRC described above.

### Data collection and outcomes

Clinicopathological data, including sex, age, previous illness, tumor characteristics, and perioperative variables were collected. Pathological staging of bladder cancer was determined in accordance with the 7th edition of the American Joint Committee on Cancer tumor–node–metastasis or TNM staging system and tumor grade was assessed in accordance with the 2004 WHO classification. The primary endpoint of this study was overall survival (OS), defined as the date of surgery to the date of death for any cause or the last follow-up. The secondary endpoints were cancer-specific survival (CSS) and recurrence-free survival (RFS). Postoperative follow-up was conducted per our institutional protocol^16^. At 3-month intervals during the first year, at 6-month intervals during the second year, and annually thereafter. We also compared the perioperative indicators, such as operating time, bleeding loss, postoperative fasting time, in-hospital mortality, and postoperative complications, among others. Complications were graded based on the Clavien–Dindo Grading System^17^.

### Statistical analysis

Categorical variables were compared using the Pearson’s *χ* ^2^-test or Fisher’s exact test. The Mann–Whitney *U* test was performed for continuous variables. Descriptive data were described as frequencies and percentages. Continuous parametric variables were described in the form of means ± SD. Nonparametric variables were presented as the median (interquartile range). To minimize the impact of treatment allocation bias, patients undergoing SRC and POPRC were propensity score-matched at a 1:1 ratio. Propensity scores were based on age, BMI, smoking status, American Society of Anesthesiologists (ASA) score, tumor size, tumor grade, pathologic T stage, and pathologic N status. The two cohorts were matched using a caliper width of 0.02 of the SD. OS, CSS, and RFS were estimated using the Kaplan–Meier method, and differences between groups were assessed using the log-rank test. A multivariable Cox regression analysis was conducted to adjust the significant confounding factors in the univariable model. The hazard ratios (HR) of OS were calculated in subgroup analyses between the two cohorts and presented using a forest plot. All statistical analyses were performed using SPSS ver. 23.0 (IBM Corp., Armonk) and the GraphPad Prism ver. 9.3.1 (GraphPad Software). A two-sided *P* < 0.05 was considered statistically significant.

## Results

### Study population

**Figure 2:**
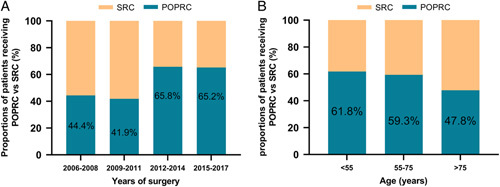
(A) The proportion of POPRC versus SRC in patients with bladder cancer at different time periods. (B) The proportion of POPRC versus SRC in different age groups. POPRC pelvic organ preserving-radical cystectomy, SRC standard radical cystectomy.

Among 2287 patients who underwent RC for bladder cancer between January 2006 and April 2018, 273 females who met the inclusion and exclusion criteria were finally enrolled in the study (Fig. 1). The median age of all patients was 69.0 (59.0–75.0) years. Among the selected patients, 158 (57.9%) underwent POPRC, and 115 underwent SRC (42.1%). The use of POPRC increased over time in a nonsignificant manner (Fig. 2A), and patients aged less than 55years are more prone to choose POPRC compared to those aged greater than 75 years (Fig. 2B). With regard to the surgical approach, the proportion of patients undergoing minimally invasive surgery increased from 33.3% in 2006–2008 to 58.9% in 2015–2017 (Supplementary Fig 1, Supplemental Digital Content 2, http://links.lww.com/JS9/A712). Table 1 summarizes the demographic and clinical characteristics of the patients before and after propensity score matching (PSM). After PSM at 1:1 ratio, 99 patients were ultimately selected for each group. The median age of the 198 patients was 69.0 (60.0–75.0) years. Compared with the patients in SRC group, more patients received orthotopic neobladder (continent urinary diversion) in the POPRC group (*P*=0.002). The other clinical and pathologic variables were well balanced between the two groups after PSM.

**Table 1:** The pre and postPSM clinicopathologic characteristics of female patients undergoing POPRC versus SRC for bladder cancer.

	Before propensity score matching			After propensity score matching		
Characteristics	SRC (*n*=115)	POPRC (*n*=158)	*P*	SRC (*n*=99)	POPRC (*n*=99)	*P*
age			0.381			0.885
≤65	42 (36.5%)	66 (41.8%)		40 (40.4%)	41 (41.4%)	
>65	73 (63.5%)	92 (58.2%)		59 (59.6%)	58 (58.6%)	
BMI	23.1±3.9	23.1±3.4	0.795	23.3±3.2	23.3±3.8	0.811
Smoking			0.380			1.000
No	108 (93.9%)	152 (96.2%)		94 (94.9%)	95 (96.0%)	
Yes	7 (6.1%)	6 (3.8%)		5 (5.1%)	4 (4.0%)	
ASA score			0.139			0.867
1–2	88 (76.5%)	108 (68.4%)		75 (75.8%)	76 (76.8%)	
3–4	27 (23.5%)	50 (31.6%)		24 (24.2%)	23 (23.2%)	
Pathologic stage			0.242			1.000
<T3	70 (60.9%)	107 (67.7%)		65 (65.7%)	65 (65.7%)	
≥T3	45 (39.1%)	51 (32.3%)		34 (34.3%)	34 (34.3%)	
Pathologic nodal status			0.559			1.000
N0	97 (84.3%)	129 (81.6%)		83 (83.8%)	83 (83.8%)	
N+	18 (15.7%)	29 (18.4%)		16 (16.2%)	16 (16.2%)	
Pathologic grade			0.315			0.576
Low	22 (19.1%)	23 (14.6%)		16 (16.2%)	19 (19.2%)	
High	93 (80.9%)	135 (85.4%)		83 (83.8%)	80 (80.8%)	
Tumor size (cm)			**0.038**			0.765
≤3	36 (31.3%)	69 (43.7%)		33 (33.3%)	35 (35.4%)	
>3	79 (68.7%)	89 (56.3%)		66 (66.7%)	64 (64.6%)	
Concomitant CIS			0.093			0.205
No	102 (88.7%)	149 (94.3%)		88 (88.9%)	93 (93.9%)	
Yes	13 (12.2%)	9 (5.7%)		11 (11.1%)	6 (6.1%)	
Positive surgical margins			0.511			0.551
No	107 (93.0%)	150 (94.9%)		92 (92.9%)	94 (94.9%)	
Yes	8 (7.0%)	8 (5.1%)		7 (7.1%)	5 (5.1%)	
Continent urinary diversion			**0.001**			**0.002**
No	108 (93.9%)	125 (79.1%)		95 (96.0%)	81 (81.8%)	
Yes	7 (6.1%)	33 (20.9%)		4 (4.0%)	18 (18.2%)	
Type of operation			0.097			0.154
Minimally invasive surgery	72 (62.6%)	83 (52.5%)		58 (58.6%)	48 (48.5%)	
Open surgery	43 (37.4%)	75 (47.5%)		41 (41.4%)	51 (51.5%)	

Categoric data are expressed as number (%) and continuous data as mean ± SD. Bold values are statistically significant (*P*<0.05). *POPRC* pelvic organ preserving-radical cystectomy. *ASA, score* American Society of Anesthesiologists score*; CIS*, carcinoma in suit; *HR*, hazard ratio; *SRC*, standard radical cystectomy.

### Perioperative outcomes

Table 2 lists the perioperative outcomes of the two cohorts. Despite more patients in the POPRC group undergoing orthotopic neobladder, the operative time in the POPRC group was shorter than that in the SRC group (*P*=0.002). Similar outcomes were observed for positive surgical margins, blood loss, postoperative fasting time, postoperative stay, surgical complications, in-hospital mortality, and 30-day mortality between the two groups.

**Table 2:** The pre and postPSM perioperative outcomes of female patients undergoing POPRC versus SRC for bladder cancer.

	Before propensity score matching			After propensity score matching		
Characteristics	SRC (*n*=115)	POPRC (*n*=158)	*P*	SRC (*n*=99)	POPRC (*n*=99)	*P*
Postoperative complications			0.182			0.233
Clavien I-II	19 (16.5%)	19 (12.0%)		15 (15.2%)	13 (13.1%)	
Clavien III-V	1 (0.9%)	6 (3.8%)		1 (1.0%)	5 (5.1%)	
Operative time (mins)	334.7±112.4	285.9±103.1	**<0.000**	338.6±113.1	287.7±99.8	0.002
Blood loss (ml)	502.9±610.6	432.7±676.1	0.461	524.4±648.5	400.0±353.3	0.767
Postoperative stay (days)	15.5±14.4	15.6±12.6	0.649	15.5±15.3	16.3±14.9	0.592
Postoperative fasting time (days)	5.1±3.5	4.9±2.5	0.877	4.8±2.6	5.0±2.6	0.526
In-hospital mortality	1 (0.9%)	0	0.873	1 (1.0%)	0	1.000
30-day mortality	1 (0.9%)	0	0.873	1 (1.0%)	0	1.000

Categoric data are expressed as number (%) and continuous data as mean ± SD. Bold values are statistically significant (*P*<0.05). *POPRC*, pelvic organ preserving-radical cystectomy; *PSM*, propensity score matching; *SRC*, standard radical cystectomy.

### Survival outcomes

**Figure 3:**
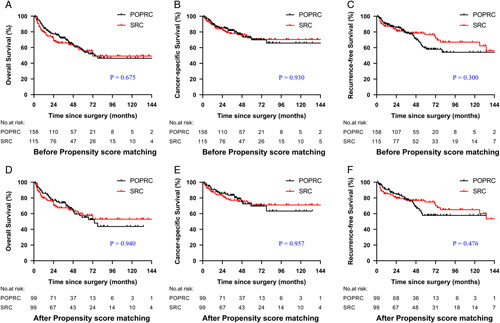
Kaplan–Meier survival curves for female patients with bladder cancer stratified by surgical methods (POPRC vs. SRC). The OS (A), CSS (B) and RFS (C) for patients before propensity score matching and the OS (D), CSS (E), and RFS (F) for patients after propensity score matching. POPRC pelvic organ preserving-radical cystectomy, SRC standard radical cystectomy, OS overall survival, CSS cancer-specific survival, RFS recurrence-free survival.

In the original cohort, the median follow-up time was 38.6 (15.9–62.5) months. A total of 109 patients (39.9%) died and 76 patients (27.8%) exhibited local recurrence or distant metastasis. Notably, no primary or secondary genital tumor was observed in patients who underwent POPRC during the follow-up period. The 5-year OS, CSS, and RFS rates were 55.1, 69.9, and 59.5% in the POPRC group versus 55.5, 70.4, and 76.6% in the SRC group, respectively. No significant differences in OS (*P*=0.675), CSS (*P*=0.930), and PFS (*P*=0.300) were determined between the two groups (Fig. 3A–C).

In the propensity-score-weighted cohort, the median follow-up time was 40.2 (17.9–66.0) months. A total of 78 patients (39.4%) died, and 59 patients (29.8%) exhibited local recurrence or distant metastasis. Kaplan–Meier survival analysis confirmed the similar long-term OS (*P*=0.940), CSS (*P*=0.957), and RFS (*P*=0.476) between the SRC and POPRC groups (Fig. 3E, F).

**Figure 4:**
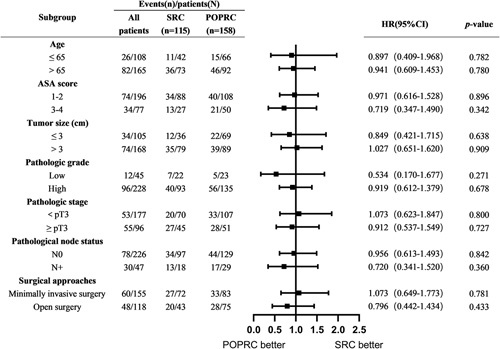
Subgroup analysis of overall survival in the original cohort. POPRC pelvic organ preserving-radical cystectomy, SRC standard radical cystectomy, HR hazard ratio, ASA American Society of Anesthesiologists.

In the exploratory subgroup analysis of OS in the original cohort, the HR of the POPRC versus SRC according to age, ASA, tumor size, pathologic grade, pathologic T stage, pathologic nodal status, and surgical approaches are presented in Figure 4. No significant difference was observed across all subgroups examined (*P*>0.05), and no heterogeneity of treatment effect was observed in these analyses (*P*_interaction_ > 0.05).

### Univariate and multivariate analyses of prognostic factors

Univariate analysis showed that age (≤ 65 vs.>65), pathologic stage (<T3 vs. ≥T3), pathologic nodal status (N0 vs. N+), pathologic grade (Low vs. High), and positive surgical margins (No vs. Yes) were significantly associated with OS. Multivariate analysis narrowed the list of independent prognostic factors for OS to age (HR 2.196, 95% CI 1.403–3.439; *P*=0.001), pathologic T stage (HR 1.776, 95% CI 1.160–2.719; *P*=0.008), and pathologic nodal status (HR 2.001, 95% CI 1.272–3.149; *P*=0.003). Surgical technique (POPRC versus SRC) was not a significant prognostic variable in the univariate (HR 0.928, 95% CI 0.635–1.356; *P*=0.700) and multivariate analyses (HR 0.874, 95% CI 0.592–1.290; *P*=0.498) (Table 3).

**Table 3:** Univariable and multivariable cox analysis of prognostic factors associated with overall survival in the original cohort.

	Univariable analysis		Multivariable analysis	
Characteristics	HR (95% CI)	*P*	HR (95% CI)	*P*
Age (years)				
≤65	1			
>65	2.388 (1.536–3.713)	<0.001	2.196 (1.403–3.439)	0.001
Smoking				
No	1			
Yes	1.501 (0.730–3.084)	0.269		
ASA score				
1–2	1			
3–4	1.420 (0.945–2.134)	0.091	1.144 (0.750–1.745)	0.533
Tumor size(cm)				
≤3	1			
>3	1.349 (0.902–2.017)	0.144		
Pathological type				
Urothelium carcinoma	1			
Nonurothelial carcinoma	1.243(0.506–3.052)	0.636		
Pathologic stage				
<T3	1			
≥T3	2.674 (1.872–3.913)	<0.001	1.776 (1.160–2.719)	0.008
Pathologic nodal status				
N0	1			
N+	2.610 (1.708–3.987)	<0.001	2.001 (1.272–3.149)	0.003
Pathologic grade				
Low	1			
High	2.281 (1.249–4.163)	0.007	1.569 (0.832–2.958)	0.164
Concomitant CIS				
No	1			
Yes	0.480 (0.196–1.177)	0.109		
Positive surgical margins				
No	1			
Yes	2.061 (1.039–4.086)	0.038	1.727 (0.845–3.531)	0.134
Continent urinary diversion				
No	1			
Yes	0.581 (0.294–1.150)	0.119		
SRC vs. POPRC				
SRC	1			
POPRC	0.928 (0.635–1.356)	0.700	0.874 (0.592–1.290)	0.498
Adjuvant chemotherapy				
No	1			
Yes	0.725 (0.378–1.390)	0.333		

Bold values are statistically significant (*P* < 0.05). *ASA*, American Society of Anesthesiologists; *CIS*, carcinoma in suit; *HR*, Hazard ratio; *N*, lymph node; *POPRC*, pelvic organ preserving-radical cystectomy; *SRC*, standard radical cystectomy.

## Discussion

Despite the use of POPRC for decades, robust evidence supporting its application in female patients remain inadequate^14^. Most existing studies involve small samples and are retrospective. To address these limitations, we comprehensively assessed a large multicenter cohort including 273 female patients with bladder cancer, assigned to either the SRC or POPRC group. In the current study, the POPRC results with respect to survival and recurrence were not inferior to those of SRC, with the proportion of POPRC increasing gradually in recent years. Our exploratory analyses also showed that the results were consistent across all subgroups examined. To our knowledge, the present study represents the largest series in the comparison of SRC and POPRC. On the basis of the studies with large samples, we performed an exploratory study for the indications of POPRC, providing evidence for patient selection of POPRC.

The main aim of RC is to cure bladder cancer and prolong patient life; meanwhile, evolving surgical techniques attempt to minimize the effect of impairment on the quality of life^18^. The primary concerns of POPRC include a potentially higher risk of positive surgical margins, local recurrences in reproductive organs, and local invasion or concomitant malignancies in gynecological organs^12^. In previous studies, bladder cancer with concomitant primary genital tumors has a relatively low probability^19,20^. However, due to selection bias and different adjuvant therapies, the rate of secondary malignant involvement of the gynecological organs is variable, ranging from 2.6–23.8%^19,21–24^. In addition, previous studies demonstrated that the factors associated with genital organ involvement included tumor location in the bladder trigone or bladder neck, clinical staging, maximum tumor size, hydronephrosis at CT, palpable mass, and positive lymph nodes^23,25,26^. However, current techniques in imaging and approaches to gynecological tumor examination (e.g. diffusion-weighted MRI, cytology-based screening, human papillomavirus-based screening, urethroscopic examination, etc.) exhibit high sensitivity and specificity for primary gynecological tumors. These qualities allow further effective screening of patients with primary or secondary cancer involving gynecological organs before surgery^27,28^. In addition, several retrospective studies demonstrated that the rate of local recurrences in reproductive organs after POPRC is low^20,23,29,30^. However, these reports consist of single-center small-sample studies. By PSM of multicenter large-sample studies, we further demonstrated that POPRC was oncologically safe in selected female patients.

POPRC is significant to improve the postoperative psychology, sexual function, and continence function of patients^12,22,31–33^. In addition, POPRC may help avoid early menopause in premenopausal women and minimize the negative effects of estrogen deficiency resulting from oophorectomy in both premenopausal and postmenopausal women. These negative effects include increased risks of coronary artery disease, stroke, Parkinsonism, dementia, depression and anxiety, bone loss, and cognitive impairment although conflicting data exist^26,33,34^. Thus, a growing number of urologists have recommended that female patients with bladder cancer choose POPRC on the basis of a thorough preoperative evaluation^12,33,35–37^. The application of POPRC increased over time in our study cohort, and young patients were more prone to choose POPRC compared to elderly patients.

With regard to elderly patients, sexual health remains an important part of life. Previous studies have suggested that patients aged greater than 55 years are not suitable for POPRC^38^. However, the sexual demands for elderly patients have raised more and more attention^39–42^. In the current study, similar oncologic outcomes between the two groups were noted in people older than 65 years old. A recent study supported our results, demonstrating that in patients over 75 years old, preservation of the anterior vaginal wall was feasible and did not compromise overall outcomes^37^. These findings suggested that POPRC might be a feasible technique among elderly patients without compromising oncologic outcomes. Moreover, POPRC can improve the postoperative sexual function and quality of life including elderly patients^12,32,33,43^.

With regard to surgical approach, previous studies have proved that minimally invasive SRC has an advantage over open surgery; meanwhile, data regarding minimally invasive surgery of POPRC were limited^32,44^. A most recent study demonstrated that robot-assisted laparoscopic POPRC is a feasible procedure associated with favorable perioperative and functional outcomes^32^. In the current study, over 50% of patients underwent laparoscopic or robotic-assisted laparoscopic POPRC and the selection of minimally POPRC increased over time. Moreover, among the patients treated with minimally invasive surgery, no significant difference in long-term survival was observed between the POPRC and SRC groups. Therefore, the minimally invasive POPRC would be a feasible choice, particularly for the non-elderly sexually and socially active women.

Several limitations need to be noted in the present study. First, this study is retrospective in nature, and selection bias may exist despite PSM by patient and tumor characteristics to reduce bias. The results obtained should be further validated in prospective studies. Second, the information about neoadjuvant chemotherapy (NAC) was incomplete due to the low rate of NAC application over the past decade in China^45,46^. However, in view of the significant pathological downstaging effect of NAC, it seems reasonable to speculate that NAC may enable more female patients to obtain the opportunity for POPRC^47–49^. Finally, sexual and urinary function data were incomplete due to the retrospective nature of this study. Therefore, we did not include these functional outcomes for comparison of surgical methods.

## Conclusion

In conclusion, no significant difference in long-term survival was determined between the female patients undergoing SRC and those undergoing POPRC. Female patients with organ-confined bladder cancer and a strong desire to preserve genital organs should be considered as candidates for POPRC and further evaluated for surgical indication.

## Ethical approval

The studies involving human participants were reviewed and approved by Sun Yat-sen Memorial Hospital, Sun Yat-sen University (SYSKY-2023-267-01); Peking University First Hospital, Peking University (2022-499-002); Peking University Third Hospital, Peking University (IRB00006761-M2023229). The patients/participants provided their written informed consent to participate in this study. Written informed consent was obtained from the patient for publication of this case report and accompanying images. A copy of the written consent is available for review by the Editor in chief of this journal on request.

## Sources of funding

This study was supported by the National Natural Science Foundation of China (Grant No. 81825016, 81961128027, 81902586, 82002679); Guangdong Provincial Natural Science Foundation (2021A1515011541, 2021A1515110200, 2023A1515010258); Guangdong Provincial Clinical Research Center for Urological Diseases (2020B1111170006); The National Key Research and Development Program of China (Grant No. 2018YFA0902803).

## Author contribution

W.Z. and J.H.: designed the study and wrote the manuscript; K.X., W.Z., L.L., S.C., P.H., W.H., W.D., H.L., Y.L., H.H., C.L., and H.Z., X.L., and G.D.: collected and analyzed the clinical data; K.X., W.Z., L.L., X.L., L.Z., T.L., and L.M.: modified and revised the manuscript; L.Z., T.L., and J.H.: supervised in the design of the study and finalized the manuscript.

## Conflicts of interest disclosure

The authors declare no potential conflicts of interest.

## Research registration unique identifying number (UIN)

1. Name of the registry: Real world-based experience of long-term oncologic outcomes of female organ-sparing radical cystectomy: a retrospective study based on medical records.
2. Unique Identifying number or registration ID: ChiCTR2000030377.
3. Hyperlink to your specific registration (must be publicly accessible and will.
4. be checked): https://www.chictr.org.cn/historyversionpub.aspx?regno=ChiCTR2000030377.

## Guarantor

Jian Huang, E-mail: huangj8@mail.sysu.edu.cn Wenlong Zhong, E-mail: zhongwlong3@mail.sysu.edu.cn.

## Data availability statement

Due to the sensitive nature of the revealed data in this study, they will not be shared. The data that has been used are confidential.

## Supplementary Material

SUPPLEMENTARY MATERIAL
