# Supplementary figures and images for: Long-term survival after female pelvic organ-sparing radical cystectomy versus standard radical cystectomy: a multi-institutional propensity score-matched analysis
Source: Int J Surg. 2023 Jun 16;109(9):2742–50. doi: 10.1097/JS9.0000000000000516 (PMC10498867; doi:10.1097/JS9.0000000000000516)

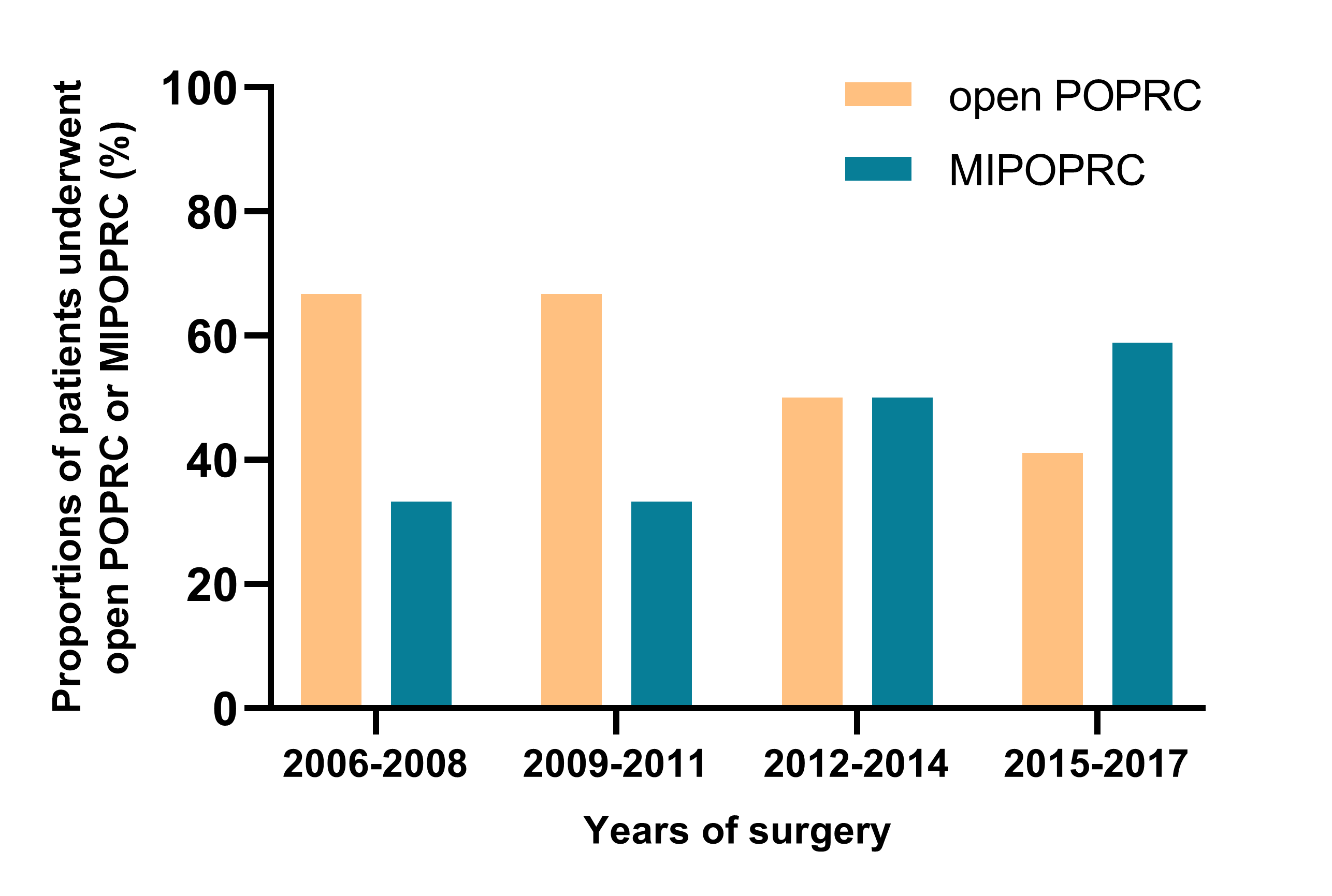

Supplement: SUPPLEMENTARY MATERIAL [file js9-109-2742-s002.tif]
